# Insulin-like growth factor-1 is a negative modulator of glucagon secretion

**DOI:** 10.18632/oncotarget.18514

**Published:** 2017-06-16

**Authors:** Elettra Mancuso, Gaia C. Mannino, Concetta Di Fatta, Anastasia Fuoco, Rosangela Spiga, Francesco Andreozzi, Giorgio Sesti

**Affiliations:** ^1^ Department of Medical and Surgical Sciences, University Magna Graecia of Catanzaro, Catanzaro, Italy

**Keywords:** glucagon, IGF-1

## Abstract

Glucagon secretion involves a combination of paracrine, autocrine, hormonal, and autonomic neural mechanisms. Type 2 diabetes often presents impaired glucagon suppression by insulin and glucose. Insulin-like growth factor-I (IGF-1) has elevated homology with insulin, and regulates pancreatic β-cells insulin secretion. Insulin and IGF-1 receptors share considerable structure homology and function. We hypothesized the existence of a mechanism linking the inhibition of α-cells glucagon secretion to IGF-1. Herein, we evaluated the association between plasma IGF-1 and glucagon levels in 116 nondiabetic adults. After adjusting for age gender and BMI, fasting glucagon levels were positively correlated with 2-h post-load glycaemia, HOMA index and fasting insulin, and were negatively correlated with IGF-1 levels. In a multivariable regression, the variables independently associated to fasting glucagon were circulating IGF-1 levels, HOMA index and BMI, explaining 20.7% variation. To unravel the molecular mechanisms beneath IGF-1 and glucagon association, we investigated whether IGF-1 directly modulates glucagon expression and secretion in an *in vitro* model of α-cells. Our data showed that IGF-1 inhibits the ability of low glucose concentration to stimulate glucagon expression and secretion via activation of the phosphatidylinositol-3-kinase/Akt/FoxO1 pathway.

Collectively, our results suggest a new regulatory role of IGF-1 on α-cells biological function.

## INTRODUCTION

Glucagon is the major hyperglycaemic hormone of the body counteracting insulin effects when blood glucose falls to dangerously low levels [1]. Glucagon secretion by pancreatic α cells is a complex and highly regulated process, involving a combination of paracrine, autocrine, hormonal, as well as autonomic neural mechanisms [2]. Glucagon secretion is mainly regulated by insulin and glucose both acting as inhibitors. Impaired suppression of glucagon by insulin and glucose have been proposed as potential mechanisms for the hyperglucagonemia observed in individuals with type 2 diabetes (T2DM) [3, 4]. Moreover, glucagon suppression upon oral glucose challenge is reduced in individuals with impaired glucose tolerance (IGT) [5–7]. However, in addition to glucose and insulin, glucagon secretion is also regulated by both the autonomic nervous system and several other hormones and metabolites including somatostatin, glucagon-like peptide-1 (GLP-1), amylin, leptin, fatty acids - which inhibit glucagon secretion; as well as glucose-dependent insulin-tropic peptide (GIP), glucagon-like peptide- 2 (GLP-2), amino-acids such as l-arginine and leucine - which stimulate glucagon secretion. Therefore, it is important to consider the complex interplay among metabolic, paracrine, autocrine, and hormonal factors when studying glucagon secretion *in vivo*.

Among hormonal factors regulating glucagon secretion, the insulin-like growth factor-I (IGF-I) is a plausible candidate. IGF-I, which has 48% amino acid sequence identity with proinsulin, is the second most powerful natural peptide with glucose lowering effects after insulin [8], and, in humans, it circulates at relatively high concentrations (150–400 ng/ml) [9]. The insulin and IGF-1 receptors share considerable homology of structure and function including common signalling pathways [10]. Lower circulating IGF-1 levels have been associated with IGT/T2DM [11–13], and insulin resistance [14, 15]. In addition, administration of recombinant IGF-1 increases glucose uptake [16, 17], and improves glucose tolerance in patients with type 2 diabetes and severe forms of insulin resistance [18–20]. By contrast, circulating IGF-1 levels are negatively associated with insulin secretion [15]. Recombinant human IGF-1 (rhIGF-1) infusion in normal subjects reduces insulin secretion while enhancing glucose disposal [21, 22], and IGF-1 exhibits inhibitory effects on glucose-induced insulin secretion in pancreatic β-cells and perfused rat pancreas [23].

However, the role of IGF-1 concentrations on glucagon secretion is less clear. The crosstalk between IGF-1 and glucagon has seldom been addressed in the literature; indeed, in the early 1990s, acute intravenous infusions of rhIGF-1 in healthy volunteers were reported to impair glucagon excretion and delay recovery after IGF-1-induced hypoglycaemia [17, 24]. Since then, no one else has attempted to verify the existence of an association between these two pivotal hormones and how IGF-1 might affect glucagon secretion *in vitro*.

We hypothesized the existence of a mechanism linking the inhibition of glucagon secretion by α-cell to IGF-1, as it has been established for β-cell secretion [23]. As a consequence, a failure to adequately suppress glucagon secretion in response to IGF-1 could contribute to hyperglucagonemia observed in prediabetes and T2DM. To address this issue, we examined the relationship between plasma IGF-1 concentrations and glucagon levels in a cohort of nondiabetic adult individuals, and tested the hypothesis that IGF-1 directly impairs glucagon expression in an *in vitro* model of pancreatic α-cells.

## RESULTS

### Cross-sectional analysis

**Figure 1:**
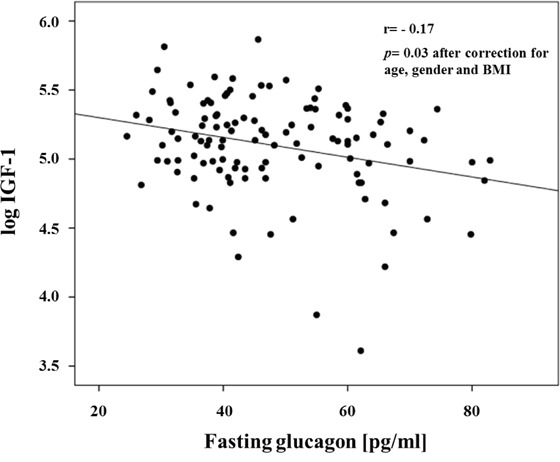
Inverse relationship between fasting glucagon and IGF-1 levels

The study cohort consisted of 116 non-diabetic subjects of whom 51 were men and 65 were women. The clinical characteristics of the whole study group are shown in Table 1. The mean age was 38.4 ±11.6 years, and mean BMI was 33.5 ± 9.8 kg/m^2^. Univariate correlations between fasting glucagon levels and anthropometric and metabolic variables are shown in Table 2. Fasting glucagon levels were significantly correlated with age, BMI and waist circumference. After adjusting for age gender and BMI, fasting glucagon levels were positively correlated with 2-h post-load glucose levels, the HOMA index of insulin resistance and fasting insulin levels, and were negatively correlated with IGF-1 levels (Figure 1 and Table 2). To estimate whether circulating IGF-1 levels were an independent contributor to fasting glucagon levels a multivariable regression analysis was performed. We found that even when age, gender and BMI, 2-h post-load glucose levels, and the HOMA index were included in the model, the variables independently associated to fasting glucagon levels were circulating IGF-1 levels (β= -0.255, *P*=0.03), HOMA index (β=0.289, *P*=0.04) and BMI (β= -0.267, *P*=0.05) explaining 20.7% of variation of fasting glucagon levels.

**Table 1:** Clinical and biochemical characteristics of study subjects

Clinical characteristic	Mean (± SD)
Gender [male/female]	51/65
Age *(yrs)*	38.4 ± 11.6
BMI *(kg/m^2^)*	33.5 ± 9.8
Waist circumference *(cm)*	109.4 ± 21.6
Systolic blood pressure *(mmHg)*	122.1 ± 15.9
Diastolic blood pressure *(mmHg)*	77.6 ± 12.1
HbA1c [%] *(mmol/mol)*	5.4 ± 0.6 [36]
IGF-1 *(ng/ml)*	173.5 ± 53.9
Fasting plasma glucose *(mg/dl)*	90 ± 11
2-h glucose *(mg/dl)*	123 ± 43
Fasting plasma insulin *(μU/ml)*	16.5 ± 11.0
Fasting glucagon *(pg/ml)*	47.7 ± 13.6
Matsuda index	64 ± 50

Data are means ± SD.

**Table 2:** Univariate correlations between fasting glucagon levels and anthropometric and biochemical variables

	Age, gender, and BMI adjusted correlations between fasting glucagon levels and metabolic variables	*P*
	Pearson's correlation coefficient (*r*)	
Age *(yrs)*	-0.19	0.003*
BMI *(kg/m^2^)*	0.14	0.05§
Waist circumference *(cm)*	0.14	0.05§
Systolic blood pressure *(mmHg)*	0.08	0.19
Diastolic blood pressure *(mmHg)*	0.01	0.48
HbA1c *(%)*	0.05	0.49
IGF-1 *(ng/ml)*	-0.17	0.03
Fasting Glucose *(mg/dl)*	0.04	0.40
2-h glucose *(mg/dl)*	0.18	0.03
Fasting plasma Insulin *(μU/ml)*	0.14	0.05
HOMA index	0.17	0.03

BMI = Body mass index; *P values refer to results after analyses with adjustment for gender. *§P* values refer to results after analyses with adjustment for age and gender.

These data suggest that circulating IGF-1 may reduce glucagon expression/secretion by pancreatic α-cells. To address this issue an *in vitro* model of pancreatic α-cells, the α-TC1 clone 6, has been used for further experiments.

### Effects of IGF-1 on preproglucagon mRNA expression

**Figure 2:**
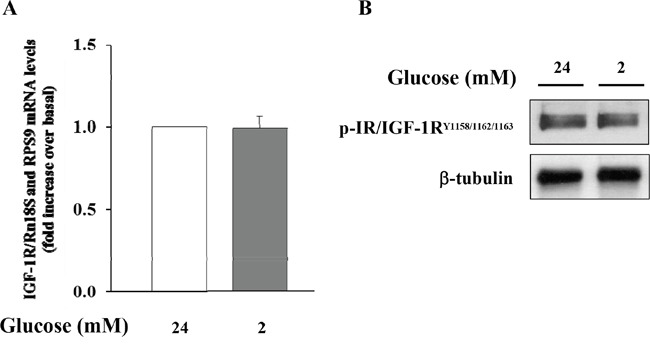
Expression of IGF-1R in α-TC1 clone 6 cultured for 24 h in DMEM under high glucose (24 mM) and low glucose (2mM) condition **(A)** The image shows Real Time RT-PCR detection of IGF-1 mRNA levels in αTC1 cells **(B)** Western blot analysis for p-IGF-1R and β-tubulin. Shown is a representative experiment for IGF-1R in α-TC1 cells.

**Figure 3:**
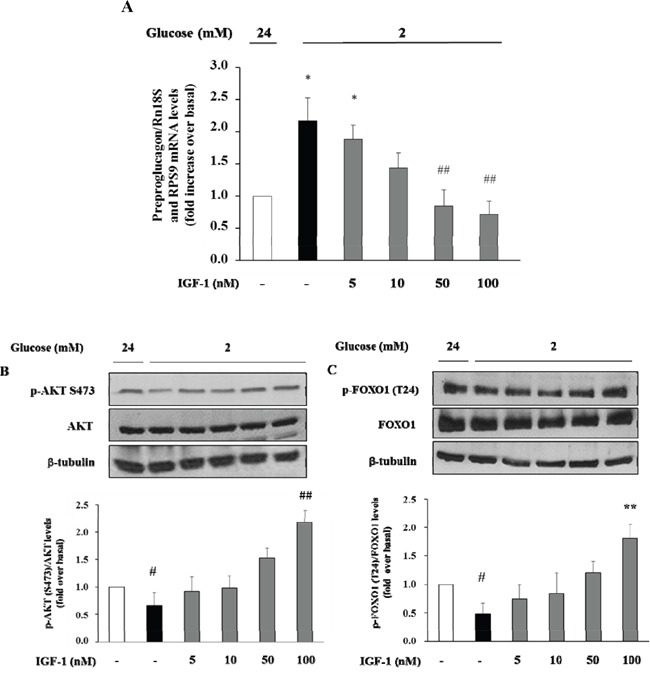
Dose-response effect of IGF-1 on preproglucagon mRNA levels, p-Akt (Ser473) and FOXO1 (Thr24) phosphorylation α-TC1 cells were incubated in presence of increasing IGF-1 concentrations (5, 10, 50, 100nM). **(A)** Preproglucagon mRNA level was measures by Real Time RT-PCR. **(B)** Representative western blot images of p-Akt (Ser473), total Akt and β-tubulin. **(C)** Representative western blot images of p-FOXO1 (Thr24), total FOXO1 and β-tubulin. Data are means ± SD of three independent experiments, each done in triplicate. *P<0.01 and ^#^P<0.05 vs. 24mM, ^##^P<0.01 and **P=0.02 vs. 2mM.

Initially, we wished to confirm the presence of the IGF-1 receptor (IGF-1R) in α-TC1 cells. The cells were cultured for 24 h in medium containing 24mM of glucose before stimulation with low glucose concentration (2mM) for 1 h. As shown in Figure 2, the IGF-1R was expressed in α-TC1 cells both in the basal state and upon low glucose concentration, as determined by Real-Time RT-PCR (Figure 2A) and Western blot (Figure 2B). Next, we evaluated the effect of IGF-1 on glucagon mRNA expression. The α-TC1 cells were incubated in absence or presence of increasing IGF-1 concentrations (5, 10, 50, 100nM) in a medium containing 24mM glucose and then were stimulated for 1 h in the presence of 2mM glucose. As shown in Figure 3, IGF-1 treatment significantly reduced preproglucagon mRNA expression induced by low glucose concentration in a dose-dependent fashion, with maximal effect occurring at 100nM (*P*<0.01).

### Effects of IGF-1 on the phosphatidylinositol-3-kinase/Akt/FoxO1 signalling cascade

**Figure 4:**
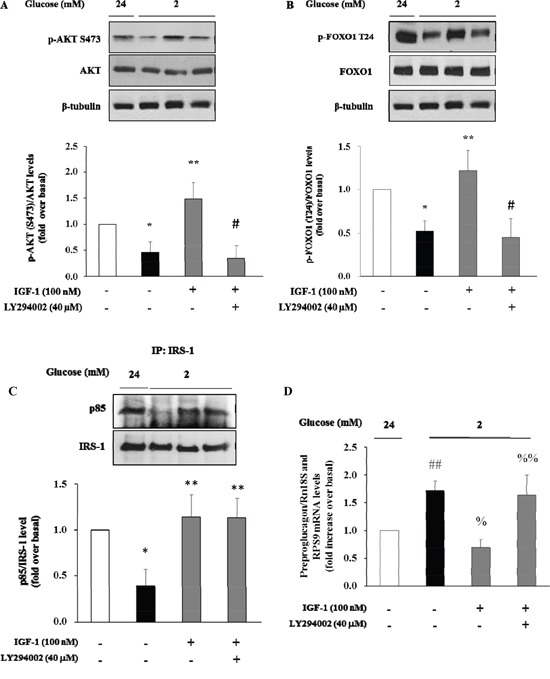
Effect of IGF-1 on PI3K/Akt pathway and preproglucagon mRNA levels α-TC1 cells were cultured with IGF-1 100nM in presence or absence of PI3K inhibitor LY294002. **(A)** Representative western blot images of p-Akt (Ser 473), total Akt and β-tubulin. **(B)** Representative western blot images of p-FoxO1 (Thr24), total FoxO1 and β-tubulin. **(C)** Representative Western blot images for p85 and total IRS-1. **(D)** Preproglucagon mRNA level measured by Real Time RT-PCR. Data are means ± SD of 4 independent experiments, *P<0.02 and ^##^P<0.01 vs 24mM, **P≤0.01 and ^%^P=0.01 vs. 2mM, ^#^P<0.01 and ^%%^P=0.02 vs. IGF-1.

There is evidence that insulin inhibits the preproglucagon gene expression by activating the phosphatidylinositol 3-kinase (PI3K)-Akt-Forkhead/winged helix box gene, group O-1 (FoxO1) signaling cascade, resulting in reduced glucagon secretion by α-TC1–9 cells [28]. Because insulin and IGF-1 share many transduction pathways including the PI3K/Akt/FoxO1 signalling cascade, we wished to inquire whether IGF-1 affected this signalling pathway in α-TC1-6. To test this hypothesis, α-TC1 cells incubated at low glucose condition (2 mM) were exposed to increasing concentration of IGF-1. Lysates from these IGF-1-treated α-TC1 cells were immunoblotted with anti-phospho-Akt (Ser473) and anti-FoxO1 (Thr24) antibody. IGF-1 increased Ser473 Akt phosphorylation 2.0-fold (basal levels vs. levels in cells stimulated with 100 nM IGF-1; P<0.01) (Figure 4A) and Thr24 FoxO1 phosphorylation 1.8-fold (basal levels vs. levels in cells stimulated with 100nM IGF-1; P<0.01) (Figure 4B). The PI3K inhibitor LY294002 is largely used to verify the effects of PI3K on downstream kinases, such as Akt, without affecting tyrosine phosphorylation of both the insulin/IGF-1 receptor beta-subunits and insulin receptor substrate 1 (IRS-1), or the association of the p85 regulatory subunit of PI3K with IRS-1 [29]. To determine whether the alterations observed in Akt and FoxO1 phosphorylation were dependent on PI3K activation, α-TC1 cells were pre-treated with LY294002 (40 μM), then the lysates were immunoprecipitated with IRS-1 antibody and blotted for p85. As reported in Figure 4C, pre-treatment with LY294002 (40 μM) markedly reduced IGF-1 stimulated Akt and FoxO1 phosphorylation, (Figure 4A and 4B, respectively). Accordingly, the inhibitory effects of IGF-1 on preproglucagon mRNA expression were abolished when α-TC1 cells were exposed to LY294002 (Figure 4D).

**Figure 5:**
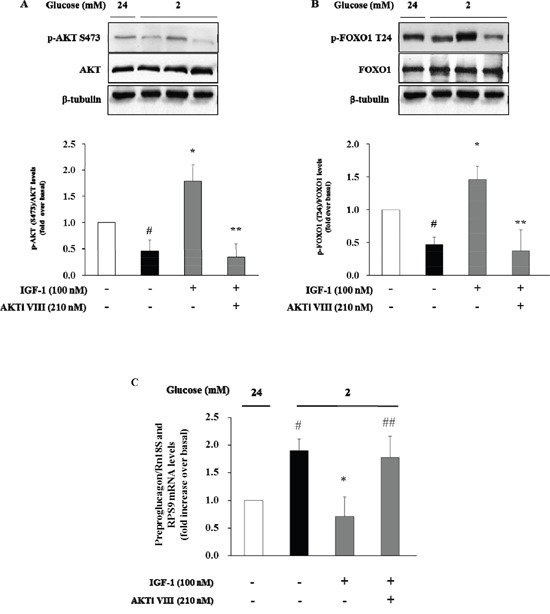
Inhibition of glucagon synthesis and preproglucagon mRNA levels by IGF-1 is Akt pathway-dependent α-TC1 cells were cultured with IGF1 100nM in presence or absence of Akt inhibitor VIII. **(A)** Representative western blot images of p-Akt (Ser473), total Akt and β-tubulin. **(B)** Representative western blot images of p-FoxO1 (Thr24), total FoxO1 and β-tubulin. **(C)** The preproglucagon mRNA level was measured by Real Time RT-PCR. Data are means ± SD of 4 independent experiments, ^#^P<0.02 vs. 24mM, *P< 0.01 vs. 2mM, ^##^p<0.02 and **P<0.01 vs. IGF-1.

We next used the Akt inhibitor VIII (AKTi VIII), a specific inhibitor of Akt, to further explore the role of Akt signalling pathway in mediating the inhibitory effect of IGF-1 on glucagon expression. To this aim, α-TC1 cells were pre-treated with AKTi VIII (210 nM), and subsequently exposed to IGF-1 (100nM), and stimulated with hypoglycemic culture medium (2mM glucose). The stimulatory effects of IGF-1 on Akt and FoxO1 phosphorylation were abolished by pre-incubation of α-TC1 cells with AKTi VIII (Figure 5A and 5B, respectively). Accordingly, the inhibitory effects of IGF-1 on preproglucagon mRNA expression were abrogated when α-TC1 cells were exposed to AKTi VIII (Figure 5C). Taken together, these results suggest that IGF-1 negatively modulates glucagon expression by activating the PI3K/Akt/FoxO1 signalling cascade.

### Effects of IGF-1 on glucagon secretion induced by low glucose concentration

**Figure 6:**
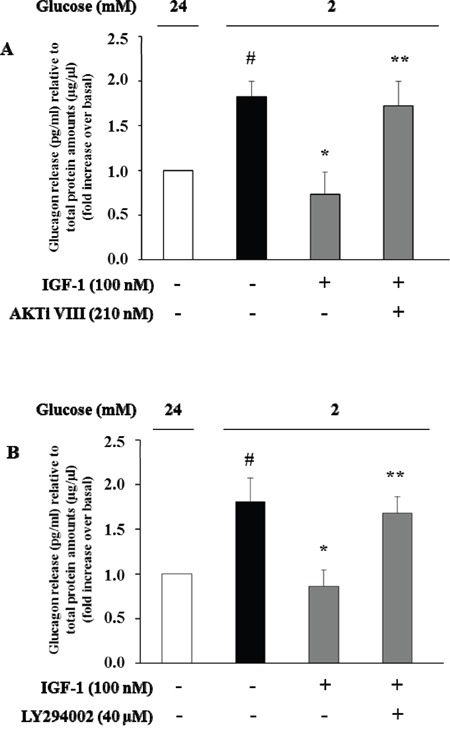
High IGF-1 concentrations inhibit glucagon secretion Histograms represent glucagon measured in the supernatants. α-TC1 cells were incubated in DMEM under high glucose (24 mM) in presence or absence of IGF-1 (100nM) and **(A)** Akt inhibitor VIII or **(B)** PI3K inhibitor LY294002. The cells were then stimulated for 1 h with low glucose (2 mM), and glucagon was measured in the supernatant and normalized for total protein amount. Data are means ± SD, of 4 experiments in triplicate, ^#^P<0.03 vs. 24mM,*P<0.02 vs. 2mM; **P<0.03 vs. IGF-1.

To evaluate the effects of IGF-1 on glucagon secretion, α-TC1 cells were exposed to IGF-1 (100nM) in medium containing 24mM glucose, and then stimulated with low glucose concentration (2mM) for 1 h. Culture media were collected and glucagon concentrations were assayed by ELISA kit. Low glucose stimulated glucagon secretion by 80% over basal (Figure 6). Treatment with IGF-1 completely abolished the stimulatory effects of low glucose concentrations. The inhibitory effects of IGF-1 were abrogated when α-TC1 cells were pre-treated with AKTi VIII or LY294002 inhibitors (Figure 6A and 6B, respectively).

## DISCUSSION

Glucagon is the paradigm of catabolic hormones, it counteracts insulin action in hypoglycaemic conditions [30], via the stimulation of gluconeogenesis and glycogenolysis in the liver [31] thus playing an important role in glucose homeostasis in both normo- and hypo-glycemic conditions. Glucagon secretion is regulated by the fine interaction of several elements, including glucose, insulin, incretin hormones, somatostatin, amino-acids, and fatty acids [31–34]. IGF-1, instead, enhances insulin metabolic and anabolic action, and lower IGF-1 levels have been shown to associate with impaired glucose tolerance, insulin resistance and metabolic syndrome, and to predict T2DM onset [15, 35–37]. The disregulation of both hormones has been suggested to contribute to the etiopathogenesis of T2DM [11, 35, 38, 38, 7].

IGF-1 is structurally and functionally similar to insulin; through the binding of its receptor (IGF-1R), IGF-1 triggers the activation of the PI3K/Akt signalling cascade, which is common to the insulin receptor signalling [39]. Convincing evidence has demonstrated that insulin signalling is an important regulator of glucagon secretion as demonstrated by studies in cellular and animal models [28, 40–42]. However, the role of IGF-1 and its signalling in modulating glucagon expression and secretion has been investigated in few reports from the 1990s. These early studies were designed to assay the immediate effects of exogenous administration of rhIGF-1 in small numbers of healthy or diabetic patients. Most of them observed a decrease in glucagon levels at different concentrations of IGF-1, despite two studies reported that basal and stimulated glucagon secretion remained unchanged [8, 43, 17, 16, 24, 44]. One molecular study from 1988 observed that glucagon release was not affected by exogenous IGF-1 in human foetal islet-like cell clusters [45]. The inconsistencies of the study designs and the small number of subjects employed for *in vivo* tests has not allowed for the hypothesis of a cross-talk between the two hormones to be resolved, yet. These observations coupled with the accessibility of a carefully characterized cohort of nondiabetic adult individuals have provided the rationale for examining the existence of a mechanism linking inhibition of glucagon secretion by α-cell to IGF-1. Here, we report an inverse relationship between circulating IGF-1 and fasting glucagon levels consistent with the pilot experimental data showing that rhIGF-1 acutely decreases glucagon concentration [22, 23]. Importantly, the inverse relationship remains significant after adjustment for several confounders potentially affecting either circulating IGF-1 or fasting glucagon levels, including age, gender, adiposity [13], insulin sensitivity [3, 15], dyslipidemia [14] and glucose tolerance status [46, 47].

The view that circulating IGF-1 is a *bona fide* modulator of glucagon secretion is supported by our *in vitro* findings in α-TC1 clone 6 cells, a mouse model of pancreatic α-cells that maintains the capability to secrete glucagon in response to low glucose concentrations. We found, for the first time, that IGF-1 dose-dependently inhibited preproglucagon expression stimulated by low glucose concentration. More importantly, these results were already observed when using physiological IGF-1 concentrations (10 nM, or 76.3 ng/ml), thus suggesting that present findings may be clinically relevant. Additionally, we show that exposure of α-TC1 cells to IGF-1 stimulates PI3K/Akt signaling, which is the canonical pathway involved in the transduction of IGF-1 action. It has been reportedly demonstrated that FoxO1 is expressed in mature primary α-cells, in which it binds directly to an upstream region within the preproglucagon gene promoter whose elimination or mutation abolishes transcriptional regulation by insulin [28, 48]. FoxO1 activity is regulated via its phosphorylation by Akt. When cells are exposed to insulin or other stimulators of the PI3K/Akt pathway, FoxO1 is phosphorylated at least at three Akt consensus sites, Thr-24, Ser-256, Ser-319. FoxO1 phosphorylation at Thr-24 results in its nuclear export and cytoplasmic sequestration through interaction with 14-3-3 proteins. Under conditions of deprivation of insulin or other stimulators of the PI3K/Akt pathway, FoxO1 is not phosphorylated at the Akt sites, and accumulates in the nucleus where it can activate genes transcription. Accordingly, we found that IGF-1 induces the phosphorylation of FoxO1 at the Thr-24 site via the PI3K/Akt pathway thus preventing the activation of preproglucagon synthesis. We support this scenario by demonstrating the cause-and-effect relationship between these two events through the inhibition of PI3K and Akt using specific chemical inhibitors of these two kinases: both inhibitors abrogate the stimulatory effects of IGF-1 on Akt and FoxO1 preventing the inhibitory effect of IGF-1 on preproglucagon expression and secretion second to low glucose concentration. It is possible to exclude the involvement of cross-interactions with the insulin receptor, because previous studies performed on murine 3T3 fibroblasts transfected with and expressing homotypic insulin receptors have shown that IGF-1 at the concentration of 100 nM interacts poorly with the insulin receptors and its signal transduction is exclusively mediated by the IGF-1R [49]. However, we cannot exclude that IGF-1 was acting through insulin/IGF-1 hybrid receptors that bind IGF-1, but not insulin, with high affinity [50, 51]. Together these data implicate PI3K/Akt/FoxO1 pathway as an important mediator of the effects of IGF-1 on preproglucagon gene expression and IGF-1 as a *bona fide* regulator of glucagon secretion. Obviously, we cannot exclude the possibility that other molecular mechanisms might mediate the inhibitory effect of IGF-1 on preproglucagon expression and secretion such as Akt-induced recruitment of the gamma-aminobutyric acid (GABA)-A receptor to the cellular membrane resulting in GABA inhibition of glucagon secretion [52]. Further studies are needed to clarify this issue.

The present study has several strengths including a larger sample size than those used in prior studies including both men and women, the measurements of metabolites and hormones in fresh blood samples rather than in stored samples that may lead to their degradation, and the centralization of biochemical assays, the exclusion of confounding conditions affecting both IGF-1 and glucagon levels, and the combination of *in vivo* and *in vitro* approaches to assess the relationship between IGF-1 and glucagon.

Nevertheless, the current study has potential limitations. First, IGF-1 and glucagon circulating levels were measured once, a common approach in clinical practice, and small variations in these variables would therefore be expected if the same assays were repeated on a different day. Second, free IGF-1 levels were not measured, and this should be considered as another limitation of the study. Moreover, in contrast with prior reports, we did not assess the acute effect of exogenous IGF-1 infusion on glucagon levels, and due to the cross-sectional design of the study, the present findings reflect only an association between circulating IGF-1 levels and fasting glucagon concentration, therefore no definitive cause and effect relationship can be inferred. Furthermore, the present study is based on outpatients recruited at a referral university hospital, representing individuals at enhanced risk for metabolic disease, and, therefore, may not be extendible to the general population. Additionally, our results are only based on White Europeans, and could not be extended to other ethnic groups. Finally, we are aware that for the assessment of α-cell function experiments in primary cells from human donors or animals would be the best option This ideal material, though, is quite rare and there is high risk of contamination with neighbouring β-cells. To overcome this issue, we have chosen to use the clone 6 derived from α-TC1 cells, which is terminally differentiated, does not express insulin, and produces glucagon in response to the hypoglycemic stimulus [53, 54]. As a safety measure in our studies we made sure to include two experimental points of basal alphaTC1-6 cells at 24 mM and 2 mM glucose concentration, in each experiment, so that we could be sure that the cells were physiologically responding to the hypoglycemic stimulus.

In summary, we provide *in vivo* and *in vitro* evidence for a role for IGF-1 signalling in the regulation of glucagon secretion by pancreatic α-cells. We propose that IGF-1 contributes to modulate glucagon secretion as *in vivo* findings recapitulate our *in vitro* results showing that IGF-1 inhibits the ability of low glucose concentration to stimulate glucagon expression and secretion via a mechanism involving activation of the PI3K/Akt/FoxO1 pathway. However, further studies are needed to clarify the inhibitory effects of IGF-1 on glucagon secretion *in vivo*, since its regulation is complex due to the combined effects of several hormones and metabolites.

## MATERIALS AND METHODS

### Study population

The study group consisted of 116 adult Caucasian subjects (age range 18-65) participating in the CAtanzaro MEtabolic RIsk factors (CATAMERI) study, an observational study assessing cardio-metabolic risk factors in individuals carrying at least one risk factor including overweight/obesity, hypertension, dyslipidemia, dysglycemia and family history for T2DM [25, 26].

Exclusion criteria were presence of end stage renal disease, chronic gastrointestinal diseases associated with malabsorption, chronic pancreatitis, history of any malignant disease, self-reporting alcohol consumption >20 g/day, positivity for antibodies to hepatitis C virus (HCV) or hepatitis B surface antigen (HBsAg), and therapy with drugs known to influence glucose tolerance (e.g. steroids, beta-blockers, and thiazide diuretics). Approval was obtained from the local ethics committee (Comitato Etico Azienda Ospedaliera “Mater Domini”) and written informed consent was obtained from each subject before commencing the studies in accordance with the principles of the Declaration of Helsinki.

### Measurements and analytical determinations

Subjects underwent anthropometrical evaluation and venous blood samples were drawn in the morning, after an overnight fast, using vacutainer tubes for laboratory determinations. Height (m) was measured to the closest centimetre, weight (kg) to the closest g, and BMI was calculated as the ratio of weight in kg to the square of height in m. Waist circumference was taken at umbilical level to the closest centimetre. Blood pressure was measured using a standard sphygmomanometer in the sitting position, as the average of the last two of three consecutive measurements obtained at 3 min intervals. After a 12-h fast, a 75-g OGTT was performed with 0, 30, 60, 90 and 120 min sampling for plasma glucose.

Glucose, triglycerides, total and high density lipoprotein (HDL) cholesterol concentrations were determined by enzymatic methods (Roche, Basel, Switzerland). Glucagon was measured using a radioimmunoassay kit (Millipore Corporation, Billerica, MA, USA). Plasma insulin concentration was measured with a chemiluminescence-based assay (Immulite^®^, Siemens Healthcare GmbH, Erlangen, Germany), and total serum IGF-1 was assayed by one-step sandwich chemiluminescence immunoassay (CLIA) after prior separation of IGF-1 from binding proteins on the Liaison® autoanalyzer (DiaSorin, Saluggia, Italy).

### α-TC1 clone 6 cells and culture conditions

Pancreatic α-TC1 clone 6 cell line (ATCC, Middlesex, UK) were grown in DMEM with L-glutamine (modified to contain 16.7 mmol/L glucose and 1.5 g/L sodium bicarbonate) supplemented with 10% (vol/vol) inactivated foetal calf serum and 1% (vol/vol) antibiotics-antimycotics, 15 mmol/L HEPES, 0.1 mmol/L nonessential amino acids, and 0.02% BSA, and maintained in a humidified atmosphere of 5% CO2 in air at 37°C. Cells were subcultured once a week and the medium was replaced at least twice weekly. α-TC1 cells were treated with IGF-1 (5, 10, 50 or 100nM) for 24 h, Akt inhibitor VIII (210nM) for 2 h and/or phosphatidylinositol-3-kinase (PI3K) inhibitor LY294002 (40μM) for 30 min, as indicated. For glucagon secretion determinations, supernatants were collected from α-TC1 cells and glucagon secretion was measured using the Glucagon Quantikine ELISA Kit (R&D Systems Europe, Lille, France) according to the manufacturer's instructions. For normalization purposes, total protein amount was determined with the Bradford assay (DC Protein Assay; Bio-Rad, Hercules, CA, USA) according to the manufacturer's instructions.

### Chemicals and reagents

Media, sera and antibiotics for cell culture were from Lonza (Walkersville, MD, USA). Akt inhibitor VIII and PI3K inhibitor LY294002 were purchased from Calbiochem (Merck KGaA, Darmstadt, Germany). IGF-1 and all other chemical reagents were obtained from Sigma-Aldrich Co. LLC (Wassergasse, Switzerland). The antibodies used were: anti-Akt, anti-p-Akt (Ser473), anti-IRS1, anti-p85, anti-FoxO1, anti-p-FoxO1 (Thr24) (Cell Signaling Technology, MA, USA), anti-p-IR/IGF-1R (Tyr 1158/1162/1163) (Merck KGaA, Darmstadt, Germany), anti β-Tubulin (Santa Cruz, CA, USA).

### Western blot analysis

α-TC1 (clone 6) protein content was obtained by lysing cells in buffer containing 50 mmol/L HEPES (pH 7.5), 150 mmol/L NaCl, 10 mmol/L EDTA, 1% Triton X-100, 10 mmol/L Na4P2O7, 100 mmol/L NaF, and 2 mmol/L sodium orthovanadate, supplemented with a cocktail of protease inhibitors. Protein concentration was determined with the Bradford assay (DC Protein Assay; Bio-Rad, Hercules, CA, USA) according to the manufacturer's instructions. Equal amounts of proteins resolved by SDS-PAGE were electrophoretically transferred to nitrocellulose membrane (Amersham Biosciences, Piscataway, NJ, USA). The membranes were incubated with primary antibodies and visualized using appropriate peroxidase-conjugated secondary antibodies followed by enhanced chemiluminescence detection (Amersham Biosciences, Piscataway, NJ, USA). Band densities were quantified by densitometry. To normalize the blots for protein levels, membranes were stripped and reprobed with anti-IRS-1, anti-Akt or anti-FoxO1 antibody after immunoblotting with their respective anti-phospho-specific antibodies. Akt and FoxO1 phosphorylation was calculated as the ratio of phosphorylated to total protein expression.

### mRNA isolation and quantification by real-time PCR

Total RNA was extracted from cells by TRIzol reagent (Life Technologies, Gaithersburg, MD, USA), according to the manufacturer's instructions. cDNA was reverse transcribed by High-Capacity cDNA Reverse Transcription Kit (Invitrogen, Thermo Fisher Scientific, Waltham, MA, USA) then analysed with TaqMan pre-designed gene-expression assays (Thermo Fisher Scientific, Waltham, MA, USA) on iQ5 real-time thermocycler (Bio-Rad, Hercules, CA, USA). Results were normalized to the geometrical average of the levels of two housekeeping genes, Rn18S and RPS9, according to the Livak method.

### Calculations

Insulin sensitivity was estimated by the HOMA index, calculated as previously described [27].

### Statistical analysis

Variables with skewed distribution including triglycerides, IGF-1, fasting insulin, and HOMA index were natural log transformed for statistical analyses. Continuous data are expressed as means ± SD. Partial correlation coefficients adjusted for age, gender and BMI were computed between variables. A multivariable linear regression analysis was performed in order to evaluate the independent contributions of plasma IGF-1 and other metabolic factors to fasting plasma glucagon levels. The variance inflection factor (VIF) was less than 2.5 in all the analyses indicating that multicollinearity among variables was not a problem in the multiple regression models with the exception of the HOMA index which showed a VIF values higher than 47 when the model included fasting glucose and insulin levels. For this reason, the model included the HOMA index rather than fasting glucose and insulin levels (VIF<2). A two-tailed P value <0.05 was considered statistically significant. All analyses were performed using SPSS (Chicago, IL, USA) software programme Version 22.0 for Windows.

## Abbreviations

IGF-1: insulin-like growth factor-1
Hb: hemoglobin
BMI: body mass index
HbA1c: glycated hemoglobin
HOMA index: homeostasis model assessment index
